# OroPress a new wireless tool for measuring oro-lingual pressures: a pilot study in healthy adults

**DOI:** 10.1186/s12984-015-0024-6

**Published:** 2015-03-24

**Authors:** Joanne McCormack, Vincent Casey, Richard Conway, Jean Saunders, Alison Perry

**Affiliations:** Department of Clinical Therapies, Faculty of Education and Health Sciences, University of Limerick, Co. Limerick, Republic of Ireland; Department of Physics and Energy, Faculty of Science and Engineering, University of Limerick, Co. Limerick, Republic of Ireland; Department of Electronic and Computer Engineering, Faculty of Science and Engineering, University of Limerick, Co. Limerick, Republic of Ireland; Statistical Consulting Unit/CSTAR@UL, University of Limerick, Co. Limerick, Republic of Ireland

**Keywords:** Oro-lingual pressure, Tongue, Isometric strength, Isometric endurance, Isometric pressure, Measurement tool, Measurement issues, Validity, Lingual, Oral

## Abstract

**Background:**

Commercially available tools for measuring oro-lingual pressures during swallowing or isometric (tongue ‘pushing’) tasks have either poor, or unknown, psychometric properties (stability, reliability) which means their validity in a clinical setting is unknown. A new wireless tool, OroPress, has been designed to address the shortcomings of existing devices. In this pilot cohort study of normal adults (i.e., people without dysphagia), the face validity of OroPress was examined when it was used to measure oro-lingual pressures during (i) isometric tongue strength (ITS) tasks and (ii) isometric tongue endurance (ITE) tasks.

The effects of gender on isometric oro-lingual data, captured using OroPress, were compared to published oro-lingual pressure data recorded using either the Kay Swallowing Workstation or the Iowa Oral Performance Instrument (aka commercial tools).

**Methods:**

Thirty five adults (17 males, 18 females), were purposefully recruited at the University of Limerick (UL), Ireland. They attended one session at the university-based clinic where their oro-lingual pressures were recorded while undertaking two isometric tasks by speech and language therapy student clinicians. OroPress was used to capture tongue strength and tongue endurance pressures during two trials of each condition and data were downloaded and analysed post-hoc. An independent-samples *t*-test and an ANOVA were used to examine the effect of gender on ITS pressures (as data were normally distributed) and an independent-samples *t*-test was used for the effect of gender on ITE pressures (where data were not normally distributed).

**Results:**

OroPress is a portable tool that was reported as being ‘easy to use’ by student SLT clinicians. The intra-oral sensor was reportedly comfortable and ‘felt non-invasive’ for participants. Data from 34 participants (16 males, 18 females) are reported.

Males did not demonstrate significantly higher mean ITS pressures than females (P = 0.057), although this approached significance, and there was no gender effect for ITE oro-lingual pressure. These results were consistent with published data from studies where other tools have been used to measure ITS pressures.

**Conclusions:**

Preliminary face validity of OroPress as a tool for recording isometric oro-lingual pressures was supported. This new wireless tool shows promise for being a criterion standard for recording oro-lingual pressures during isometric tasks.

## Background

During swallowing, the tongue is essential for generating the major propulsive force on a bolus [1], so it is important to examine tongue control as part of a comprehensive swallowing assessment in people who have dysphagia (impairment in swallowing). Clinically, speech and language therapists (SLTs) use subjective methods to examine lingual control, e.g., by judging the force a patient applies when pushing their tongue against a tongue depressor held by the clinician [2]. Such assessments have no established validity (the degree to which the tool measures what it is intended to measure [3]) or reliability (the extent to which a test or measurement is reproducible [4]), so their accuracy and clinical utility may be questioned. For this reason, researchers prefer to assess lingual control by examining isometric (pushing against a resistance) strength (force) and/or endurance (ability to sustain force over time) of the tongue, using instrumental oro-lingual pressure measurement devices [2].

Two of the most widely used commercially available devices are (i) tongue bulb arrays attached to the Kay Swallowing Workstation (KSW) and (ii) the Iowa Oral Performance Instrument (IOPI) [5]. The KSW is a computerised system with either a two or three-bulb array–i.e., air-filled sensors embedded in a silicon strip-that may be used synchronously with a videofluoroscopic swallowing study (VFSS) to record oro-lingual pressure. The three sensor array is adhered to the hard palate and records anterior, medial, and posterior oro-lingual during either isometric or swallowing tasks [1,2,5-10]. The IOPI is a hand-held, portable pressure transducer, consisting of a single air-filled plastic bulb (typically held behind the alveolar ridge by clinician or client), connected to a liquid crystal display via a plastic tube and is used for oro-lingual pressure measurement during isometric tasks [11-15]. More recently, the Madison Oral Strengthening Therapeutic (MOST) device (a custom-fit mouthpiece with multiple sensors-see Table 1) [16] has been developed to record oro-lingual pressure. These devices have been used to examine the effect of isometric tongue strength (ITS)- i.e., recorded maximal oro-lingual pressure reached during approximately one second of resistance [13] on an efficient and/or safe swallow [11-15,17]. By contrast, examining isometric tongue endurance (ITE)- i.e., maximum oro-lingual pressure sustained over an extended time period [13]; as a representative measure of tongue function has received little attention [12].

**Table 1:** **A comparison of the IOPI, KSW& MOST tools for oro-lingual pressure measurement**

**Item**	^**a**^ *KSW tongue array	^**b**^^**†**^ **IOPI**	^**c**^^**‡**^ **MOST**
Visualisation of signal	Excellent	Good	Good
Quantitative data	Yes (optional)	Yes	Yes
Swallow Environment	Lab only	Clinic/Lab	Clinic/Lab
Sensor Array	3 air filled sensors (hand-held & fixed position versions)	1 liquid filled sensor (hand-held)	Custom-fit mouthpiece with four sensors
Ease of use	Needs skilled user	Easy to use	Reported to be easy to use
Data Integrity	Good-With fixed sensors	Poor–movement artefacts	Good
Intrusiveness	Intrusive-gag risk	Intrusive and fragile	Intrusive and effect normal swallow
Patient usage	Fixed position	Hand held device	Fixed position
Pressure Data	Swallowing, isotonic	Isotonic only	Swallowing, isotonic
Costs:			
(a) Hardware	(a) ^d^ **€80-90k	(a) ** €4k	(a) Not available
(b) Probes	(b) **€40/single use	(b) ** €10/single use	(b) Not available

^a^ * KSW: Kay Swallowing Workstation.

^b^^**†**^ IOPI: Iowa Oral Performance Instrument.

^c^^**‡**^ MOST: Madison Oral Strengthening Therapeutic device.

^d^ ** €: Euro.

Despite a growing body of published research based on data taken from the IOPI, KSW [1,2,5-12,18-21], and/or the MOST [16] all three tools are acknowledged to have a number of disadvantages which include: having limited clinical utility, partly due to poor reliability of data capture (IOPI); [1,16,22] the lack of stability of the sensor position (IOPI); [5] the intrusiveness of the probes (all tools); [17,19,20,22] the high cost of the single-use sensors (all tools); the (lack of) portability of the device (KSW); the high cost of hardware (KSW) and the IOPI cannot be used to capture pressure measurements when swallowing food or fluid boluses [5]. See Table 1 for a comparison of these tools.

To address these shortcomings, we have produced a wireless device, OroPress. This is a new oro-lingual pressure measurement system which may be used for capturing and recording both isometric and swallowing oro-lingual pressure data. We have identified a new parameter-isometric tongue endurance (ITE) or Pt100, which is the pressure–time product for the region where the oro-lingual pressure is maintained above 100 mmHg during the isometric tongue endurance task. This is an improvement on the previously used ITE measure, where participants maintained 50% of their maximal pressure for as long as possible [13]. Such a measure only provides information about the duration of the trial, so does not provide a comprehensive measure of the overall effort exerted (both duration (time) of trial and strength of pressure). In contrast, Pt100 represents the ‘area’ of the ITE pressure waveform, so is a comprehensive index of the tongue propulsion index (in mechanical terms–the impulse).

This study is the first of a sequence to examine the clinical utility, safety and psychometric properties of OroPress. In this pilot study, measurements of oro-lingual pressures generated during isometric strength and endurance tasks were investigated. The face validity (i.e., the extent to which an instrument appears to test what it is intended to test) [3] of OroPress as a tool for measuring oro-lingual pressures was examined by comparing the effects of gender on norm ITS and ITE pressures captured with OroPress with published data where the KSW and/or the IOPI were used. Although we were able to do this for ITS data (Table 2) we were unable to do this for ITE data as there was limited published research into the effects of gender on ITE pressures, and different ITE parameters were used in the present study to analyse ITE data than those in the literature.

**Table 2:** **Reported gender differences in maximum isometric tongue strength pressures (PmaxS) of normal adults**

**Author(s)**	**Device**	**Sample size**	Male: PMaxS*	Female: PMaxS*	**Sig. diff in gender**
Vitorino, 2010 [21]	IOPI	N = 75	56.81 ± 7.21 kPa	56.37 ± 6.9 kPa	Yes
		(m = 35, f = 40)			
Stierwalt & Youmans, 2007 [10]	IOPI	N = 200	63.24 ± 13.86 kPa	57.15 ± 13.50 kPa	Yes
		(m = 80, f = 120)			
Youmans & Stierwalt, 2006 [2]	IOPI	N = 90	64.0 ± 13.7 kPa	55.9 ± 12.5 kPa	Yes
		(m = 45, f = 45)			
Crow & Ship, 1996 [11]	IOPI	N = 99	74.8 ± 18.9 kPa	64.7 ± 19.6 kPa	Yes
		(m = 52, f = 47)			
Nicosia et al., 2000 [5]	KSW	N = 20	Not provided	Not provided	No

*Maximum pressure during an isometric tongue strength task.

[Mean and standard deviations are reported].

We hypothesised that (i) females would demonstrate significantly lower ITS pressures than males; (ii) there would be no significant gender differences for ITE recordings, as a recent systematic review indicated that gender had a significant effect on ITS pressure (males demonstrating significantly higher ITS pressures than females), but gender had no effect on ITE pressures [23].

## Methods

### Participants

Participants were recruited from advertising posters and verbal requests across the campus of UL. A total of 35 normal healthy adults were purposefully recruited (17 males, 18 females).

Participants attended the Speech and Language Therapy (SLT) clinic at UL where they provided informed consent and were then screened with a short questionnaire about their past and present swallowing function. Exclusion criteria included: having a history of a swallowing and/or speech disorder; having a medical condition, or use of medications, which affect swallowing. An oro-motor examination excluded people with any oral abnormality and those with an overly sensitive gag reflex (i.e., gag reflex triggered in the middle portion of the surface of the tongue). People who were unable to give informed consent or to follow oral instructions were also excluded. Approval for this study, conforming to the Helsinki Declaration, was obtained from the University of Limerick’s Faculty of Education and Health Sciences’ Research Ethics Committee.

### OroPress system

**Figure 1:**
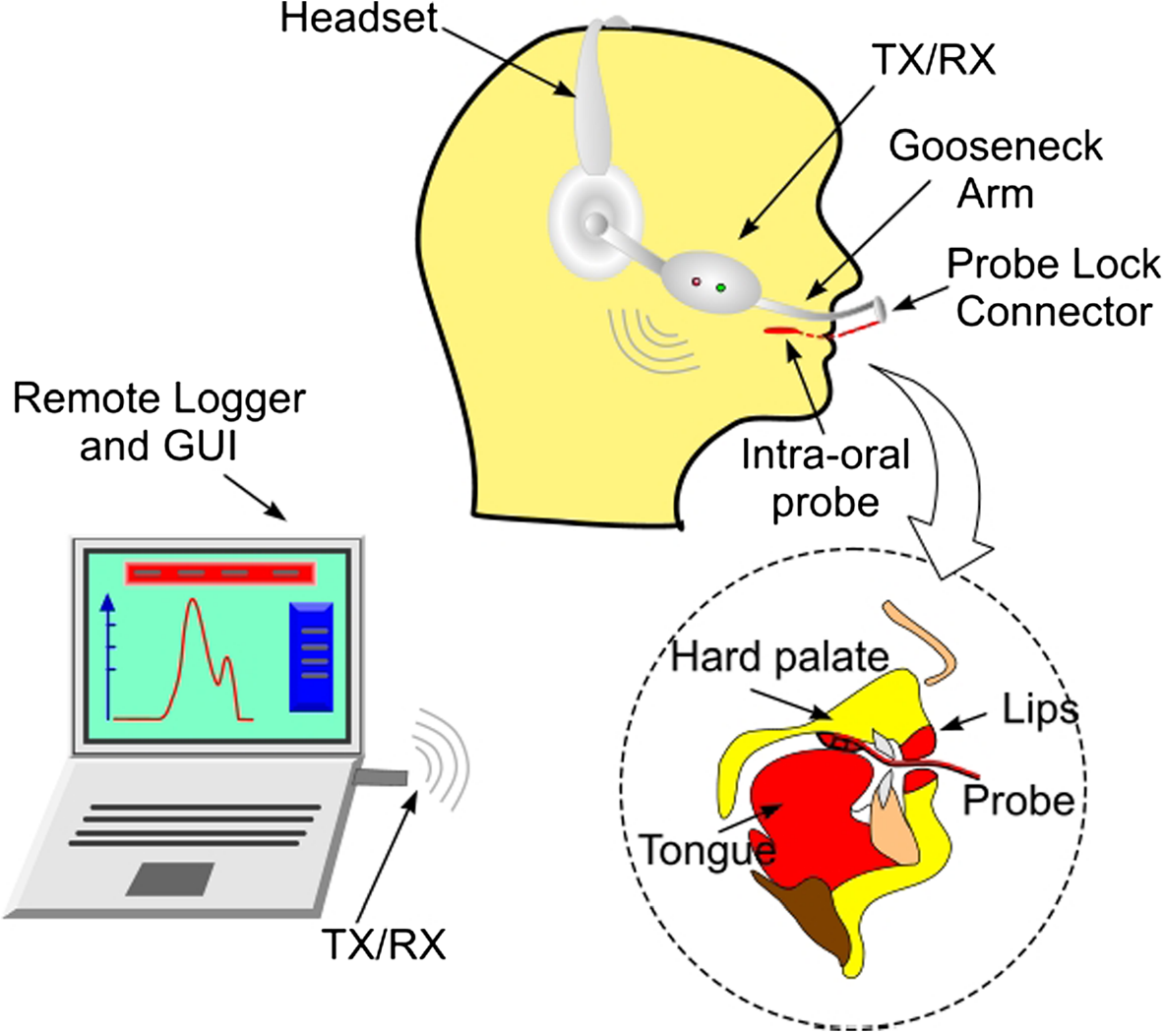
**The components of OroPress.**

The OroPress system consists of a Biomedical Interface Pressure Transducer (BIPT-i.e., a sensor) [22], a headset and a wireless transmission module which transmits data to a remote laptop or notebook for real-time display and recording (Figure 1). The OroPress sensor measures the pressure applied by the tongue directly at the site of the sensor-tongue interface (rather than indirectly through a column of air or fluid as with other tools).

**Figure 2:**
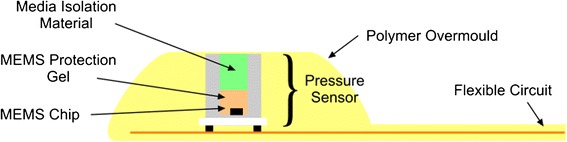
**Cross-sectional representation of OroPress sensor.**

The BIPT is 3 mm in height and approximately 16 mm in diameter with a plano-hemispherical profile containing a digital pressure sensing module (MS58 series, Measurement Specialities Ltd., Switzerland) with accuracy over the range 0–225 mmHg of approximately 1 mmHg. The pressure sensing module has been modified to enable it to be used for measuring both tissue contact/interface pressures and bolus pressure. The micro-electromechanical system (MEMS) chip is protected by a layer of soft MEMS protection gel. The gel is coated with a non-stick media isolation material (Figure 2). This combination ensures that the module can be used to measure both fluid/semisolid bolus pressure and tissue interface pressure, e.g., tongue applied pressure, without being destroyed or contaminated. The media isolation material and gel faithfully relay the pressure at the interface to the MEMS chip [24].

In use, the planar surface faces the hard palate/alveolar-ridge and the contoured hemispherical surface containing the pressure sensing area faces the tongue (Figure 2). The body of the BIPT is formed from a soft polymer which allows a degree of conformation to curved surfaces and reduces the intrusiveness of the device. A planar flexible cable that allows normal mouth closure and lip movement was used to connect the BIPT to the headset unit.

**Figure 3:**
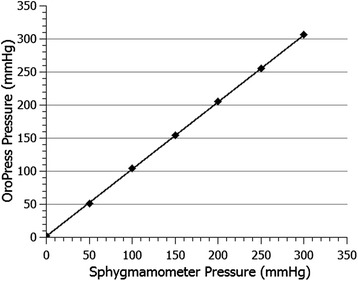
**A graphical representation of the calibration of the OroPress sensor.**

A Zigbee transceiver from Telegesis was used with the microcontroller to transmit measurements wirelessly to a remote notebook computer. Zigbee enabled low power operation over a range up to 20 m and provided full security, including data authentication and privacy, in addition to complete electrical isolation between OroPress (on the participant) and a mains-powered notebook. The remote computer displayed the received measurements in real-time using a 2-dimensional pressure–time graph which updated every 10 milliseconds, i.e., 100 samples per second. Optional recording of data to file was also provided (Figure 1). A mercury sphygmomanometer was used to check sensor response over a pressure range of 0-300 mmHg (0–40 kPa). Sensor calibration adjustment was not required during the study duration (over 3 months), indicating very good long-term sensor stability (Figure 3). The student clinicians were trained in the use of the device (both the software and hardware) and rehearsed approx. 10 ‘trial runs’) of the study protocol prior to study commencing.

### Data collection

**Figure 4:**
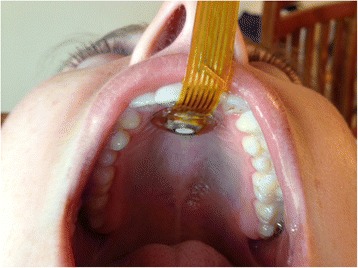
**Positioning of OroPress sensor for measuring oro-lingual pressure.**

After receiving informed consent and screening a participant he/she was seated 2 metres from a facing wall where a disc was placed at their eye-level. Participants were instructed to gaze at the disc during data collection to ensure that their head was stabilized in a neutral position, i.e. neither looking upwards nor downwards. The SLT in adhered the small OroPress sensor (Figure 2) to a participant’s alveolar ridge (hard palate) using a Poligrip ComfiSeal strip (Figure 4). Once the sensor was in-situ, each participant first practised the oro-lingual pressure tasks (both strength and endurance) without being recorded, to become accustomed to the sensor. Once they stated they were comfortable, trials commenced (two trials for each condition). The order of oro-lingual pressure tasks were counterbalanced for strength versus endurance, to control for possible effect of learning and/or fatigue on pressure generation.

#### Isometric tongue strength task

Each participant was instructed to, *‘push the tip of your tongue as hard as possible against the sensor for three seconds after I say go.’* The trial was timed with a stopwatch and participants were instructed to, ‘stop pushing’ at three seconds.

#### Isometric tongue endurance task

Each participant was instructed to, *‘push the tip of your tongue as hard as you can against the sensor, for as long as you can when I say go.’* The data captured on the laptop were observed and participants were instructed to ‘stop pushing’ once any significant dip in oro-lingual pressure (i.e., below a 100 mmHg threshold) was noted. A minimum pressure marker of 100 mmHg was used to identify the beginning and end of a stable ITE response, in order to ensure that the oro-lingual pressures captured were ‘true’ readings and not due to extraneous variables, such as a participant’s tongue inadvertently probing the sensor. By choosing 100 mmHg as the threshold, we avoided capturing such artefacts of measurement.

#### Study design

**Figure 5:**
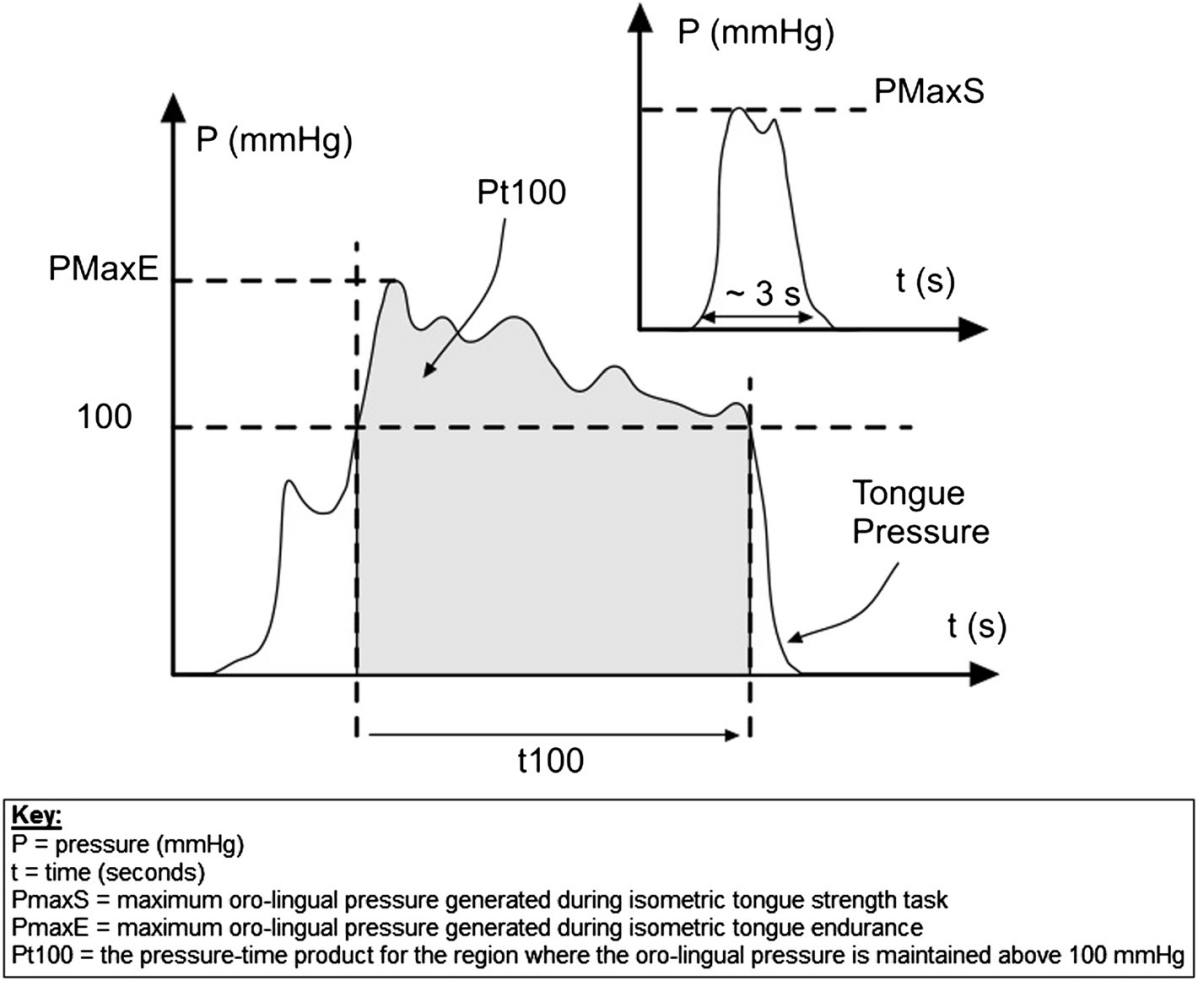
**A graphical representation of the parameters used to measure isometric tongue strength and isometric tongue endurance.**

This was a pilot cohort study with one independent variable (IV), gender (male, female), and four dependent variables (DVs), (i) PmaxS [mmHg]–the maximum oro-lingual pressure generated during the isometric tongue strength (ITS) task (Figure 5); (ii) PmaxE [mmHg]-the maximum oro-lingual pressure generated during the isometric tongue endurance (ITE) task (Figure 5); (iii) t100 [seconds]–the time for which the oro-lingual pressure is maintained above 100 mmHg (13.3 kPa) during the ITE task; (iv) Pt100 [mmHg s]-the pressure–time product for the region of the graphed results where the oro-lingual pressure is maintained above 100 mmHg (Figure 5). The latter measurement, Pt100, corresponds to the area under the pressure–time plot between the time where the pressure first rises above 100 mmHg and first drops below 100 mmHg and, as such, it provides a representative index of the tongue propulsion index (in mechanical terms–the impulse).

### Data management

Data from the OroPress were transferred to a Microsoft Excel spreadsheet, together with de-identified participant data (identification number, age and gender). Any recorded oro-lingual pressure under 100 mmHg was not included in analyses.

#### Data extraction

All of the study data was entered into a Microsoft Excel spreadsheet for preliminary review. A LabView (National Instruments UK & Ireland, Newbury, Berkshire, UK) virtual instrument was developed to extract the pressure peaks, in order to compress the data.

Peaks from participants’ data were then each copied to a single Excel worksheet and simple formulae were used to extract the parameters of interest. The four calculations (DVs) included: the maximum oro-lingual pressure generated during the isometric tongue strength (ITS) task (PmaxS, Figure 5); the maximum oro-lingual pressure generated during the isometric tongue endurance (ITE) task (PmaxE, Figure 5); the time for which oro-lingual pressure is maintained above 100 mmHg (13.3 kPa) during the ITE task (t100, Figure 5) and the pressure–time product for the region where the oro-lingual pressure is maintained above 100 mmHg during the ITE task (Pt100, Figure 5).

#### Data screening

Data screening included an examination of means, medians and standard deviations, analysing box plots and histograms, calculating skewedness and kurtosis, and applying the Shapiro-Wilk test to ensure data conformed to assumptions of normality [25]. Once confirmed, parametric testing was applied to the ITS data. As ITE pressure data were not normally distributed, non-parametric testing was used.

#### Statistical analysis

The data, extracted using the algorithms as above, were entered into IBM® SPSS® Version 20 software package [26] for statistical analyses. The data underwent a series of steps in analysis. The influence of gender on ITS pressures obtained with OroPress was examined using an independent-samples *t*-test and ANOVA, adjusting for age. The effect of gender on ITE pressures (obtained with OroPress) was examined using an independent-samples *t*-test.

#### Descriptive statistics

Descriptive statistics were used to examine the characteristics of the sample [3]. For ITS (PMaxS) and for ITE (PMaxE, t100, Pt100) data included measures of central tendency, such as the mean and median, and measures of variability, such as range and standard deviation [3].

## Results

Data from one male were excluded as the quality of his recorded pressure waveforms was poor, due to researcher error during data capture. Results from 34 norm participants (m = 16; f = 18) are therefore here reported.

### Descriptive statistics

Normally distributed data (PMaxS and PMaxE; Table 3) were given means and confidence intervals (CI’s), and skewed/not normal data (t100 and Pt100; Table 4) were given medians and inter-quartile ranges (IQR’s). Male participants achieved a higher mean PMaxS (574 mmHg i.e., 76.49 kPa) than females (488 mmHg i.e., 65.09 kPa). This difference (p = 0.057) was non-significant, although it approached significance (Table 3). Applying an ANOVA adjusting for age as well as gender, the sex difference was still not significant (p = 0.09) but the power of this test was only 39.7%, suggesting that this result may reach significance with a larger sample size.

**Table 3:** **Gender differences for normally distributed parameters-PmaxS and PmaxE**

	**Mean (mmHg)**	**SD**	**95% CI**
PMaxS (all)	528.48	131.54	428.37, 574.37
Males	573.74	143.11	497.49, 650.00
Females	488.24	108.96	434.06, 542.43
PMaxE (all)	547.52	142.59	497.76, 597.27
Males	556.20	138.33	482.49, 629.91
Females	539.80	149.83	465.29, 614.31

*Abbreviations:* PMaxS, maximum oro-lingual pressure generated during isometric tongue strength task; PmaxE: maximum oro-lingual pressure generated during the isometric tongue endurance task; SD, Standard deviation; CI, Confidence interval.

**Table 4:** **Gender differences for not-normally distributed parameters-t100 and Pt100**

	**Median**	**IQR**
t100 (all)	^a^ *11.43	8.70, 17.88
Males	*10.25	7.96, 16.80
Females	*13.15	9.89, 20.50
Pt100 (all)	^b^^†^4407.25	3448.35, 5775.60
Males	^†^4246.10	3347.28, 6015.93
Females	^†^4407.25	3629.98, 5775.60

*Abbreviations:* t100, the time for which the oro-lingual pressure is maintained above 100 mmHg during the isometric tongue endurance task; Pt100, the pressure–time product for the region where the oro-lingual pressure is maintained above 100 mmHg during the isometric tongue endurance task; IQR, Inter-quartile range.

a. *Seconds.

b. ^†^mmHg/Sec.

Males produced a stronger mean score for PMaxE (556 mmHg) than females (540 mmHg) but the difference was not significant (p = 0.743) (Table 3). From the t100 measure, females had longer median t100 durations (13 seconds) than males (10 seconds) but this difference was not statistically significant. On exploring Pt100 data, female participants demonstrated larger median Pt100 pressure areas (4,407 mmHg/sec) than males (4,246 mmHg/sec); although again the difference was non-significant (Table 4).

### Within subject reliability

Further data was collected from 35 normal healthy adults (m = 19; f = 16) using OroPress after the data for the present pilot study was completed. Each participant performed three ITS and three ITE tasks. Within subject reliability was examined using the Intraclass Correlation Coefficient (ICC) which “reflects both correlation and agreement” [3]. For the PMaxS measurement the ICC agreement was 0.861 and for the PMaxE measurement, 0.687.

## Discussion

Results of this pilot study provide important information about a new wireless tool, OroPress, when used to measure oro-lingual pressures. Preliminarily results suggest that males have higher ITS pressures than females (Table 3), which is consistent with findings from some previous researchers (Table 2-in particular Crow and Ship, 1996 [11]); however, the difference was not significant (P = 0.057), perhaps reflecting the small sample size. ITS pressure data differentiated males from females, giving preliminary face validity for this new tool.

Three newly developed parameters isometric tongue endurance (ITE); the maximum ITE pressure (PMaxE); time for which ITE pressure is maintained above 100 mmHg (t100); and the pressure–time product for the region where ITE pressure is maintained above 100 mmHg (Pt100); were examined for differences in ITE between males and females. Of these, Pt100 is the most robust measurement as it quantifies the total effort made for the endurance interval. This will, for example, distinguish between two participants who have similar PMaxE and similar t100, but have different average or sustained pressures for the t100 period. Pt100 may thus provide a better indicator of the overall effort expended by a participant in the endurance task. There was evidence of a gender difference for ITE, with females having larger Pt100 pressure areas, but this difference was again not statistically significant (Table 4). A possible explanation for these findings is that the male participants exerted more lingual effort/strength to produce the higher PMaxE (maximal pressure) than the females during the ITE trials. Consequently, males may have fatigued faster [27,28], as demonstrated by their shorter t100 (duration/time) and a smaller Pt100 (area); but, further research into the effect of gender on ITE pressures is needed to confirm this.

To examine within subject reliability, further data were collecting using OroPress after the present pilot study was completed. Thirty five normal healthy adults (m = 19; f = 16) performed three ITS and three ITE tasks and an ICC was used to examine within subject reliability. ICC for the PMaxS measurement was 0.861 which is indicative of good reliability as it is above 0.75, and the ICC for the PMaxE measurement was 0.687 which suggests moderate to poor reliability as it is below 0.75 [3]. Such findings provide indicate that OroPress is a very reliable tool for measuring PMaxS.

### Study limitations

The results of this pilot study are valuable for future studies of OroPress. The small sample size (n = 35) in this pilot study is acknowledged, resulting in low statistical power to establish group differences, and the exclusionary criteria may have reduce the sample’s representativeness [4,29], but work is continuing to build a larger norm data set. From this study we now have data to conduct a power calculation to estimate the sample size required to assess gender differences for example, in ITS pressures captured using OroPress. A sample size of 51 in each group will have 90% power to detect a difference in means of 85.5 (the difference between a Group 1 mean, μ_1_, of 573.74 and a Group 2 mean, μ_2_, of 488.24) assuming that the common standard deviation is 131.54 using a two group *t*-test with a 0.050 two-sided significance level. Alternatively, a sample size of 39 in each group will have 80% power to detect a difference in means of 85.5 (the difference between a Group 1 mean, μ_1_, of 573.74 and a Group 2 mean, μ_2_, of 488.24) assuming that the common standard deviation is 131.54 using a two group *t*-test with a 0.05 two-sided significance level. From this study there is evidence of OroPress’s face validity for measuring oro-lingual pressure. The reliability and construct validity (the degree to which a tool reflects its theoretical foundations or measures what it is intended to measure) [4] can now be examined.

## Conclusions

From this pilot study, a sound testing protocol for OroPress was established its preliminary face validity for measuring oro-lingual pressures was demonstrated and a sample size calculation for future studies has been conducted. With its novel wireless sensor, OroPress offers a more accurate and stable oro-lingual pressure measurement system, using a low profile sensor design for data capture. Improved characterisation of ITE by examining the area (Pt100) of the captured ITE waveform is now possible. Further work using OroPress to develop a larger data set of norm oro-lingual (swallowing and isometric) pressures against which clinical populations can be assessed is ongoing. We anticipate OroPress will be the criterion standard measurement tool to use with clinical populations (aka adults with dysphagia).

## Abbreviations

ITS: Isometric tongue strength
ITE: Isometric tongue endurance
IOPI: Iowa oral performance instrument
KSW: Kay swallowing workstation
PmaxS: Maximum oro-lingual pressure generated during isometric tongue strength task
PmaxE: Maximum oro-lingual pressure generated during the isometric tongue endurance task
t100: The time for which the oro-lingual pressure is maintained above 100 mmHg during the isometric tongue endurance task
Pt100: The pressure–time product for the region where the oro-lingual pressure is maintained above 100 mmHg during the isometric tongue endurance task
